# Value of brain MRI in infective endocarditis: a narrative literature review

**DOI:** 10.1007/s10096-015-2523-6

**Published:** 2015-11-19

**Authors:** J. Champey, P. Pavese, H. Bouvaist, A. Kastler, A. Krainik, P. Francois

**Affiliations:** Medical Intensive Care Department, CHU de Grenoble, BP 218, 38043 Grenoble Cedex 9, France; Infectious Diseases Department, CHU de Grenoble, BP 218, 38043 Grenoble Cedex 9, France; Cardiology Department, CHU Grenoble, Grenoble, France; Neuroradiology Department, CHU Grenoble, Grenoble, France; Public Health Department, CHU Grenoble, Grenoble, France

## Abstract

The nervous system is frequently involved in patients with infective endocarditis (IE). A systematic review of the literature was realized in accordance with the Preferred Reporting Items for Systematic Reviews and Meta-Analysis (PRISMA). This study sought to systematically evaluate the published evidence of the contribution of brain magnetic resonance imaging (MRI) in IE. The aim was to identify studies presenting the incidence and type of MRI brain lesions in IE. Fifteen relevant studies were isolated using the Medline, Embase, and Cochrane databases. Most of them were observational studies with a small number of patients. MRI studies demonstrated a wide variety and high frequency of cerebral lesions, around 80 % of which were mostly clinically occult. This review shows MRI’s superiority compared to brain computed tomography (CT) for the diagnosis of neurologic complications. Recent developments of sensitive MRI sequences can detect microinfarction and cerebral microhemorrhages. However, the clinical significance of these microhemorrhages, also called cerebral microbleeds (CMBs), remains uncertain. Because some MRI neurological lesions are a distinctive IE feature, they can have a broader involvement in diagnosis and therapeutic decisions. Even if cerebral MRI offers new perspectives for better IE management, there is not enough scientific proof to recommend it in current guidelines. The literature remains incomplete regarding the impact of MRI on concerted decision-making. The long-term prognosis of CMBs has not been evaluated to date and requires further studies. Today, brain MRI can be used on a case-by-case basis based on a clinician’s appraisal.

## Introduction

Despite significant improvements in diagnosis and therapeutic strategies, infective endocarditis (IE) is still a serious disease. The in-hospital mortality rate remains high, at around 20 % [1].

Among systemic complications, the central nervous system is the first involved and it depends on one anatomic substratum: septic emboli arising from bacterial vegetation fragmentation [2]. With an incidence rate of around 30 % and a 30 % associated mortality rate, neurological damage is a major concern for clinicians [3–5]. Neurological symptoms are delayed, frequent, and often subclinical [3]. They can be varied, including focal deficit, encephalopathy, and seizures. Most often, these symptoms call for early brain computed tomography (CT). Furthermore, systematic neuroimaging for all patients, even those without clinical neurologic symptoms, is now considered routine. The significance of asymptomatic cerebral lesions is the root cause of undertaking imaging studies. A quick and accurate neurologic assessment seems essential to rapidly determine IE treatment. Mainly with cardiac surgery, clinicians aim to control cardiac involvement, decrease embolic risk, and control widespread infection. Magnetic resonance imaging (MRI) has been shown to be more sensitive and more specific than CT in this indication. However, the role of MRI has not been accurately defined.

The goal of the present review was to summarize the current data concerning the indication for brain MRI in IE management. This review details the incidence of MRI lesions and the characteristics of each type of lesion. Then, the value of MRI in IE diagnosis, the choice of treatment, and prognosis evaluation will be discussed.

## Research question and search strategy

This review was established using the Preferred Reporting Items for Systematic Reviews and Meta-Analysis (PRISMA) guidelines [6]. The Medline and Cochrane databases and Embase were searched from January 1994 to February 2014.

The search strategy is described in Table 1. The results of searches were stored in an Excel database. The search terms used in the Cochrane library and in Embase were the same as those used in PubMed. Firstly, 224 references were documented and stored in the Zotero personal research assistant. References of full texts were also reviewed in order to identify any potentially relevant study.

**Table 1:** Search strategy applied in the Medline, Embase, and Cochrane databases

Search performed in the following numerical order:
#1 infectious endocarditis
#2 infective endocarditis
#3 magnetic resonance imaging
#4 brain
#5 cerebrovascular disorders
#6 microbleeds
#7 #1 OR #2
#8 #7 AND #3
#9 #7 AND #4
#10 #7 AND #5
#11 #8 AND #5
#12 #7 AND #6
#13 #8 AND #6

Studies reporting the incidence and types of brain MRI lesions in IE were included. Because of the shortage of studies, we selected articles regarding brain MRI and endocarditis with broad characteristics, but we only retained studies with a major criterion on the value of brain MRI. The decision to retain an article was made based on the title, the abstract, and the complete article. Data were extracted by two reviewers. The selection was limited to studies on hospitalized adults and studies published in English. Retrospective studies were retained in the analysis. Articles using CT scanning of the brain as a comparator were retained. Studies were excluded if they included fewer than ten participants or if the endocarditis cases were not diagnosed in accordance with the modified international Duke criteria as definite or probable IE.

## Results

### Study selection and characteristics

**Fig. 1:**
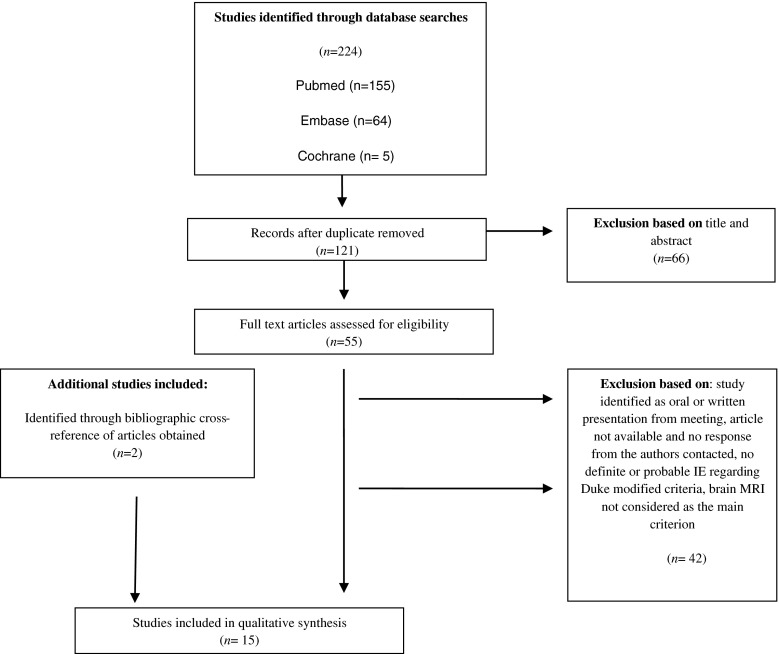
Flow chart of the study selection process

Fifteen clinical studies were found that were suitable for inclusion in the review. Among the full-text articles available, no response was received from the authors contacted in one case. Figure 1 summarizes the article acquisition. The studies’ characteristics are listed in Table 2. The quality of the studies was analyzed using the STROBE Statement and the PRISMA checklist. Most of them were transversal studies, cross-over studies, or retrospective studies. No randomized controlled trials or comparative studies were obtained. The rarity of the disease, its polymorphous features, and the numerous prognostic factors may explain the infrequency of randomized studies on IE. Most of the time, sample sizes were small and all the patients included did not undergo brain MRI. Seven prospective studies were identified. Most of them had the highest number of patients and required all our attention [7–10].

**Table 2:** Summary of the included studies

Study	Study period	Inclusion criteria	Design of study	Number of participants	Mean age	Imaging protocol	Main results	MRI follow-up	Neurocognitive follow-up
Morofuji et al., 2010, Clinical Neurology and Neurosurgery, Japan [26]	2006–2007	IE^a^ diagnosis according to modified Duke criteria	Retrospective monocentric	11	54	T2*- weighted MRI^b^ protocol	72.7 % of patients had MRI^b^ abnormalities; 63.2 % of patients showed CMBs^c^; number of CMBs^c^ increased in 14–28 days in five patients with multiple lesions	In the month after initial MRI^b^ for seven patients	No
Singhal et al., 2002, Stroke, USA [14]	1993–2000	Definite or possible IE^a^ according to Duke criteria	Retrospective monocentric comparison of acute and recurrent ischemic stroke in IE^a^ and NBTE^d^	35 (27 IE/9 NBTE^d^)	53	DWI^e^ MRI in the second week after the onset of neurological symptoms	NBTE^d^ patients uniformly had multiple widely distributed, small and large strokes, whereas IE^a^ patients exhibited also other patterns, such as a single lesion, territorial lesion, disseminated punctate lesions; disseminated punctate lesions were related to clinical encephalopathy	In the month after initial MRI^b^ for four IE^a^ patients	No
Hess et al., 2013, AJNR. American Journal of Neuroradiology, France [7]	2005–2008	Definite or possible IE^a^ according to Duke criteria	Prospective monocentric	109	59	MRI^b^ imaging within 7 days after inclusion GRE^f^ and DWI^e^ sequences; double blinding interpretation	MRI^b^ showed abnormalities in 71.5 % of patients; 37 % ischemic lesions; 57 % CMBs^c^; eight patients had acute hemorrhage, three had microabscesses, three had small cortical hemorrhage, three had mycotic aneurysm; 52.5 % of lesions had different ages; 62.5 % of ischemic lesions were multiple small infarcts disseminated in watershed territories; CMBs^c^ were preferentially distributed in cortical areas; no significant relation was found between ischemia and CMBs^c^	No	No
Klein et al., 2009, Stroke, France [22]	2005–2008	Definite or possible IE^a^ according to Duke criteria	Retrospective case–control study	60 IE^a^ matched with 120 controls	62	Cerebral MRI^b^ within 7 days after admission; standardized protocol GRE^f^ and DWI^e^ sequences; double blinding interpretation	CMBs^c^ were more prevalent in IE^a^ patients (57 %) than in control subjects [15 %; odds ratio (OR) 10.06]; the OR of IE^a^ increased dramatically with the number of CMBs	No	No
Goulenok et al., 2013, Cerebrovascular Diseases, France [1]	2005–2007	Probable or definite IE^a^ according to modified Duke criteria and neurological complications	Prospective monocentric cohort study	30	58	Cerebral MRI^b^ within 7 days after admission; MRI^b^ comparison with non-contrast CT^g^ (*n* = 5 ) and angio-CT^g^ (*n* = 26); imaging review by a blinded neuroradiologist	MRI^b^ findings: ischemia (*n* = 25), CMBs^c^(*n* = 17), mycotic aneurysm (*n* = 7), abscess (*n* = 6), subarachnoid hemorrhage (*n* = 5), vascular occlusion (*n* = 3), hemorrhagic lesion (*n* = 2); in 19/30 cases, neurologic symptoms were observed before IE^a^ diagnosis; none of the 16 operated patients underwent postoperative worsening; MRI^b^ was more sensitive than CT^g^ in detecting both symptomatic (100 and 81 %, respectively) and silent cerebral lesions (50 and 23 %, respectively); therapeutic plans were modified according to the MRI results in 27 % of patients, including surgical plan in 20 %	No	No
Iung et al., 2013, Stroke, France [20]	2005–2008	Definite or possible IE^a^ according to Duke clinical criteria	Prospective monocentric cohort study	120	61	Cerebral MRI^b^ within 7 days following admission	MRI^b^ detected ischemic lesions in 53.3 % of patients and CMBs^c^ in 60 % of patients; ischemic lesions were associated with vegetation length and *Staphylococcus aureus*; CMBs^c^ were associated with no prior anticoagulant therapy and prosthetic IE^a^; vegetation length >4 mm identified ischemic lesions with 74.6 % sensitivity and 51.5 % specificity		
Cooper et al., 2009, Circulation, USA [9]	2004–2007	Definite IE^a^ according to modified Duke criteria in patients in whom there was at least one left-side heart valve involvement on echocardiography	Prospective	56	58	Brain MRI^b^ as soon as possible and before any surgical intervention; imaging review by a blinded neuroradiologist	80 % acute brain embolization; 48 % subacute brain embolization; lower risk of mortality at 3 months with cardiac surgery; OR 0.1 (0.003–0.6), *p* = 0.008	No	No
Okazaki et al., 2013, Cerebrovascular Diseases, Japan [16]	2004–2011	Only definite left-side IE^a^	Retrospective multicentric (six university hospitals)	85 (47 MRI^b^)	58	Preoperative operative DWI^e^ and fluid attenuated inversion recovery sequences MRI^b^ within 14 days after diagnosis; imaging reviewed by an experienced neurologist in clinical blinding; only few patients underwent T2* GRE^f^ sequences; no microhemorrhages investigation	55 % of patients had acute ischemic lesions, 60 % had small lesions, 77 % had multiple lesions, 64 % had lesions in multiple vascular territories; plasma CRP^h^ level and white blood cell count were associated with ischemic lesions (ORs 2.3 and 2.2, respectively); no associations were found between postoperative complications and preoperative acute ischemic lesions; only three patients underwent postoperative neurologic complications; mean time from MRI^b^ to cardiac surgery was 22 days	No	No
Iung et al., 2012, European Heart Journal Cardiovascular Imaging, France [8]	2005–2008	Definite or possible acute IE^a^ according to modified Duke criteria and “excluded” IE^a^ with high clinical suspicion	Prospective monocentric; diagnostic classification and therapeuticplans establishment by two experts before and after MRI^b^; comparison of the two assessments	58	61	Cerebral and abdominal MRI^b^; standardized protocol; double blinding; MRI^b^ <7 days of diagnosis; GRE^f^ and DWI^e^ sequences	Based on MRI^b^ results and excluding CMBs^c^: 19 % modified therapeutic plans, 28 % modified diagnostic classification	No	No
Funakoshi et al., 2011, The Journal of Thoracic and Cardiovascular Surgery, Japan [34]	2006–2010	Active native valve IE^a^ with surgical indication	Retrospective monocentric	18	53	Preoperative angio-MRI^b^; GRE^f^ and DWI^e^ sequences	Urgent surgery for 15 patients; among them, 10 (67 %) showed IE^a^ brain lesions, ten patients had acute or subacute brain infarctions, two had brain infarction with abscess, and two had hemorrhagic brain infarction and did not go on to have surgery	No	No
Jeon et al., 2010, Cerebrovascular Disease, Korea [25]	2005–2006	No previous cardiac surgery and elective cardiac surgery	Prospective monocentric	45 (19 MRI^b^)	53	Preoperative and postoperative GRE^f^ and DWI^e^; MRI^b^; standardized protocol; double blinding; interpretation	26 new postoperative GRE^f^ lesions in 12 patients	Four patients	No
Snygg-Martin et al., 2008, Clinical Infectious Diseases, Sweden [10]	1998–2001, 2002–2005	High clinical suspicion of left-side IE^a^	Prospective bicentric	60 (49 MRI^b^)	63.5	Brain MRI^b^ or CT^g^ scan <10 days of antibiotics; cerebrospinal fluids analyses of inflammatory and neurochemical markers of brain damage; no DWI^e^ MRI sequences	65 % cerebrovascular complications, 30 % were silent	No	No
Duval et al., 2010, Annals of Internal Medicine, France [11]	2005–2008	Definite or possible acute IE^a^ according to modified Duke criteria and “excluded” IE^a^ with high clinical suspicion	Prospective monocentric; cohort study; two experts jointly established the endocarditis diagnostic classification and therapeutic plans just before and after MRI^b^ and then compared them	130	59	Cerebral MRI^b^ within 7 days after admission and before any surgical intervention; double blinding; MRI^b^ <7 days of diagnoses; GRE^f^ and DWI^e^ sequences	82 % MRI^b^ abnormalities; diagnostic classification of 32 % of cases of indefinite IE^a^ was upgraded in 18 % of therapeutic plans, modifications including 14 % surgical changes	No	No
Grabowski et al., 2011, J of Neurology, Poland [8]	2002–2008	IE^a^ diagnosis according to modified Duke criteria without prevented neuroimaging examinations or evident hemorrhagic stroke	Prospective monocentric	65 (52 MRI^b^)	49	MRI^b^ or CT^g^ Imaging	Of 65 patients: 13 patients with a clinical neurologic event, 24 patients with silent embolism (46 %), a total of 37 patients with neurologic lesions (56.9 %)	No	No
Azuma et al., 2009, Japanese Journal of Radiology, Japan [17]	2004–2006	IE^a^ with clinical neurologic complications	Retrospective monocentric	14	70.4	Cerebral MRI^b^ within 9 days following admission; only four T1 gadolinium imaging; DWI^e^ only for 13 patients; T2* GRE^f^ only for three patients	Thirteen patients had cerebral lesion embolization: in ten patients, most often in multiple territories, mainly in cortical and subcortical areas for nine; 57 % of cases involved the middle cerebral artery and 42.9 % of cases concerned the posterior cerebral artery; three cases of intracranial bleeding, three abscesses, two cerebritis; four patients presented CMBs^c^; half of the patients had more than two abnormal MRI^b^ findings	No	No

^a^Infective endocarditis

^b^Magnetic resonance imaging

^c^Cerebral microbleeds

^d^Non-bacterial thrombotic endocarditis

^e^Diffusion-weighted imaging

^f^Gradient recalled echo

^g^Computed tomography

^h^C-reactive protein

Since the literature concerning infectious aneurysm and brain abscess MRI detection was rare, case studies and reviews on these topics were also analyzed. These articles were considered separately and were not included in Table 2.

### Incidence of lesions

The use of brain CT had previously shown high overall incidence of cerebral lesions, but this incidence was even higher with the use of MRI [1].

Including 130 patients, the largest MRI study reported 82 % cerebral lesions, among which 78 % were asymptomatic [11]. The radiological data quality was high using a standardized protocol and double blinding by two experienced neuroradiologists with a high interobserver concordance. All patients promptly underwent MRI within 7 days of admission.

The studies by Snygg-Martin et al. and Cooper et al. reported 65 % and 80 % brain MRI abnormalities with, respectively, 35 % and 48 % silent lesions as well [9, 10]. Nonetheless, no diffusion-weighted images were performed in the study showing the lower values.

Because all studies excluded patients who underwent emergency surgery before any MRI, the exact incidence remains unknown and the overall prevalence could be underdiagnosed.

Most CT scans with undetected lesions and justifying systematic MRI use were microhemorrhagic lesions and small ischemic strokes [1]. Small infarcts were detected in 53 % of cases and MRI identified cerebral microbleeds (CMBs) in 57 % of cases [7, 8]. As for microlesion detection, the importance of MRI sequences in interpreting the study results should be noted [12]. Radiological criteria guidelines for CMB detection and differential diagnosis have not been precisely established [13]. In this sense, the uniformity of the neuroimaging protocol and complete assessment using gradient recalled echo (GRE) and diffusion-weighted imaging (DWI) sequences seems necessary to correctly highlight small infarctions and numerous CMBs in IE. Furthermore, other lesions are less common (parenchymal hemorrhage, subarachnoid hemorrhage, abscess, and aneurysm) and have the same incidence with MRI as with CT.

### Types of lesion

#### Ischemic lesions

With CT studies, it has previously been demonstrated that ischemic stroke is the major neurological complication of IE. Most patients show multiple lesions with various patterns [6, 13]. DWI sequences detected very acute and very small ischemic lesions [14]. The loss of hyperintensity in the DWI signal with time is also helpful to differentiate acute and chronic infarction. Infarct can be infra- and supratentorial [14]. A recent neuroradiologic study showed 40 % infratentorial damage [7]. The specific severity of a posterior fossa bleeding transformation should be considered in the therapeutic decision.

MRI also demonstrated a neuroimaging embolism spectrum. Several years ago, Singhal et al., Barsic et al., and Okazaki et al. noted the predominance of smaller lesions [14–16], which, instead, affect cortical and border zone arterial territories [17]. Similarly, Hess et al., on 109 prospective cases without neurologic symptoms, emphasized the frequency of acute ischemic lesions and of small infarcts widespread in watershed territories [7]. In this sample, most patients had multiple bihemispheric lesions lodged in the supratentorial gray–white junction. This confirms widespread cardiac emboli. Nevertheless, IE stroke patterns are polymorphic [14]; a single lesion or territorial infarction suggesting a solitary embolus is also possible. Regarding the arterial territories, cortical branch infarction was the most common lesion, which usually involved the distal middle cerebral artery tree [14].

Valvular vegetation size correlated with the size and number of infarcts [18, 19]. Moreover, a recent and large series found a 10 % increase in the toll of ischemic lesions for every millimeter increase in vegetation length [20].

Because the structure and function of *Staphylococcus* cells enable metastatic diffusion, *S. aureus* etiology is frequently correlated with emboli [10, 21]. Other embolic risk factors are well known: mitral valve reach, mobile vegetation length >10 mm, and systemic embolism [20].

#### Cerebral microbleeds

In MRI, T2*-weighted GRE sequences are planned to see small foci of hemorrhages, also called microbleeds. CT is not able to detect CMBs. This hypointense T2*-weighted MRI round or ovoid signal with a diameter <10 mm can be detected in elderly patients and, overall, in small-vessel disease such as arteriolosclerosis, chronic hypertension, amyloid angiopathy, as well as Alzheimer’s disease [13, 17]. They notably have a strong specific IE association. In a case–control study of 60 cases, CMBs were more prevalent in IE patients (57 %) than in control subjects [15 %, matched odds ratio (OR): 10] [20]. The atherosclerosis factor was similar in the two groups.

CMBs seem to indicate a pattern of vascular vulnerability and were previously described as a potential indicator of hemorrhagic stroke in IE. This study was a 26-patient retrospective study with low methodological weight [16]. While several analyses found that CMBs might predict future risk of symptomatic intracerebral hemorrhage, a number of cardiac surgery studies did not show an additional risk of postsurgical bleeding [9]. If necessary, anticoagulation is still beneficial [12, 13].

CMBs have a higher incidence in IE than in other cerebral diseases and likely a specific distribution [7, 22]. This specific topography has been demonstrated in two studies, but only one of them was prospective and described IE CMBs; the mean number of CMBs was five [23, 24]. They were preferentially distributed in cortical areas (85 %) and were mostly large and heterogeneous. They are less frequently located in subcortical white matter basal ganglia or posterior fossa than in the general population. In contrast with previous data, in this study, no significant relation was found between macrohemorrhage and CMBs. CMBs were only associated with prosthetic IE and not with prior anticoagulant therapy. Fundamentally, no relationship between CMBs and ischemic lesions or emboli was highlighted. It can be hypothesized de facto that CMBs might be due to the severity of infection itself. CMBs may represent pyogenic vasculitis or subacute inflammatory microvascular process [1]. Hyperintense T2* halo and T1 enhancement is also a specific inflammatory characteristic of IE CMBs. Indeed, inflammatory marker levels are higher in cases of CMBs [12], which may also reflect a relation with infection. To this day, CMB etiology and significance remain unclear.

In view of supporting the use of these characteristic cranial MRI lesions as an additional IE diagnostic marker, the underlying pathologic process needs to be better known. The predictive factors must also be understood. However, various other elements have to be confirmed; one study showed a rise in the number of CMBs after cardiac valve surgery in 19 patients. New CMBs were found in about two-thirds of the patients, but these lesions seem to be different from those found in preoperative IE [25]; they may be a consequence of mechanical valve microfragmentation. Some genetic factors have also been related to CMBs, most particularly in amyloid angiopathy disease [12, 13].

The recurrence, enhancement, and degradation of these subclinical lesions is unknown because radiological progression studies are lacking. In the only study with 11 patients who underwent a follow-up brain MRI, the number of IE CMBs increased in comparison with the initial MRI, suggesting an active process [26].

#### Hemorrhagic lesions

Cerebral bleeding includes subarachnoidal bleeding (SAH), lobar hemorrhages, and hemorrhagic transformation of an ischemic stroke. Acute SAH is well detected on gradient recalled fluid attenuated inversion recovery (FLAIR) sequences, whereas SAH sequelae are seen on T2* sequences [20]. Compared to CT, MRI showed a limited value for lobar hemorrhages and infarction secondary bleeding diagnosis.

Among the different causes of parenchymal hemorrhage and SAH, a ruptured mycotic aneurysm or suppurated necrotizing focal arteritis must be removed by a systematic angiography sequence.

#### Intracranial microbial aneurysms

Microbial aneurysms can be seen with time-of-flight weighted imaging, angiography, and three-dimensional T1-weighted imaging after gadolinium enhancement.

Even through no recent large series have been conducted, magnetic resonance angiography is as effective as angio-CT in diagnosing microbial aneurysms. They both have approximately 95 % sensitivity for the diagnosis of an aneurysm with a diameter greater than 5 mm [23, 24].

The diagnosis of small aneurysms remains difficult and, until now, conventional angiography is the best choice to ensure a complete and exact diagnosis. This technique allows aneurysm evaluation and treatment.

Mycotic aneurysms are usually multiple, bilateral, distal, and fusiform, but the angiographic presentations can vary widely [25, 27]. The middle artery territory is usually involved, especially the distal tree [7, 24]. Often, vascular occlusion or stenosis can be associated. Coiling may be required as preventive treatment before any cardiac surgery or as curative treatment in case of non-aneurysm involution under suitable antibiotic therapy.

#### Brain abscess and meningitis

A macroabscess is typically seen as an expansive lesion with central restricted apparent diffusion, hyperintense peripheral edema on the FLAIR sequence, and post-contrast annular enhancement [8]. In IE, numerous microabscesses are more frequent and often smaller than 1 cm. Even with the MRI contribution, it is not always straightforward to differentiate microabscesses and septic embolism, most particularly for small and presuppurative lesions.

Regarding meningeal irritation, lepto- or pachymeningeal contrast enhancement as a non-specific T2 FLAIR hyperintense signal of the subarachnoid is more readily and clearly seen with MRI than with CT. Such imaging enhancement is more limited in clinical practice.

## Discussion

### Main results

The present review confirms that MRI is more sensitive than CT in brain damage detection. Most lesions undetected on CT and justifying systematic MRI are small hemorrhagic lesions and widespread microinfarcts. A significant proportion of these brain lesions are clinically occult.

### MRI timing

Recurrent strokes can be clinically silent and mostly occur during the first week of antibiotic therapy [3]. Brain imaging must be performed as early as possible to quickly optimize IE management [26]. Because neurologic worsening occurs during the acute phase of any IE, clinicians must plan for neuroimaging screening. Ideally, MRI should be performed in the few days after diagnosis. MRI timing obviously depends on technical support availability and the radiologist’s approval.

### Brain MRI: an additional diagnostic criterion

IE diagnosis is difficult and frequently delayed, with an average time to diagnosis of 30 days [28]. In some insidious presentations, fever can be absent. Echocardiography and blood cultures may be falsely negative. Particularly in the elderly or among hemodialysis patients or in case of intracardiac devices, the Duke criteria can be long to acquire. These criteria were initially developed for clinical research, and certain clinical practice limitations remain. This diagnosis period, without treatment, is a source of damage progression and sequelae.

With know-how, infectious diseases specialists can treat patients with major suspicion of IE but without complete Duke criteria, like an IE. MRI might provide additional diagnostic clues. Excluding CMBs, a recent large study suggested that cerebral MRI, performed up to 7 days after admission, changed the diagnosis classification in 32 % of cases [11]. Including CMBs, they upgraded the diagnoses in 51 % of patients with initially non-definite IE.

Moreover, with Hess et al.’s neuroradiologic precision, the pattern of silent ischemic lesions and CMBs lesions could become a further diagnostic marker [7]. Additional studies are needed for CMBs to be used as an IE feature and consider them as a new minor imaging criterion for IE.

### Brain MRI in the surgical decision

Although cardiac surgery recommendations in circumstances of severe congestive heart failure or persistent severe infection are clear, embolic arguments for surgery remain more ambiguous [15]. It is not always easy to assess the benefit–risk ratio of an early surgery. Surgical timing is sometimes controversial, most particularly a major hesitation for immediate valvular surgery in patients with high embolic risk and recent stroke. However, if cerebral bleeding or major ischemia is a transient surgical contraindication, cardiac surgery can be safely performed after silent cerebrovascular complications [21]. In cases of embolic risk factors associated with small MRI lesions, there is no benefit in delaying surgery [7].

To date, several surgical studies have shown contradictory results regarding valvular surgery at the acute phase of IE [29–32]. In patients with a high embolic risk, there seems to be a benefit of early surgery in terms of mortality, with less risk of new symptomatic events [21, 33, 34]. Therefore, MRI results and complete neurological assessment should lead clinicians to change the surgical strategy with regards to the indication for cardiac surgery and timing of valve replacement.

A prospective study in 120 IE patients has recently demonstrated that MRI can impact early surgical management. Excluding CMBs and solely on the basis of MRI results, Duval et al. showed modifications in surgical plans in 18 % of cases [11]. Moreover, brain MRI may be fitted into a complete imaging checkup. Some teams perform brain MRI, a chest, abdomen, and pelvis CT scan, and echocardiography on the same day, at an early stage, to thoroughly map asymptomatic lesions.

In summary, most postponement surgeries result from a fear of postoperative neurological aggravation. On the contrary, when there are no obvious contraindications, the early removal of the primary infectious site (cardiac vegetation) is the best way to impede neurologic injury. In this view, brain MRI could help make a decision for early surgery. The optimal therapeutic strategy may vary in individual patients and a multidisciplinary team in each case should discuss the timing of surgery.

### Value of brain MRI antibiotic modification

Brain MRI findings may also lead to specific medical cure modifications. Although meningitis, abscesses, and empyema can be seen on CT, MRI is particularly useful in diagnosing small lesions. In some infectious disease departments, microischemia or CMBs are viewed as a trigger for antibiotic adjustment. Central nervous system infections require antimicrobial drugs with high cerebral diffusion. Changes can be an increase in dosage, a switch, or addition of another drug. A 7 % change was found in the only study aiming for antibiotic therapy regimen modifications with the use of systematic brain MRI [11].

The obvious continuum between infection and emboli in IE may support the clinicians’ decision. Indeed, IE brain complications can be suppurate, inflammatory, or solely ischemic lesions [7]. Especially concerning CMB etiology, we do not have robust data and there is no evidence to treat such lesions like microabscess. In addition, the prognosis of brain parenchyma diffusion of an antibiotic used after brain MRI has not been evaluated.

### Prognosis evaluation

When symptomatic, cerebral damage argues for a worsening prognosis [5], although only moderate to severe ischemic stroke and brain hemorrhage were significantly associated with a poorer prognosis. Silent lesions do not carry a significant excess mortality [21].

Concerning CMB implication, the association between cognitive impairment, disability, and CMBs has been described in non-IE diseases, such as Alzheimer’s disease [25]. In IE, the long-term prognosis of CMBs and small ischemic lesions has never been evaluated. Few longitudinal studies have been conducted. These vasculitis patterns might only be a marker of sepsis severity.

### MRI: practical use and limits

Currently, with progress in medical implants, there are fewer and fewer contraindications to MRI. Although MRI may still be limited in some instable patients, improvements have made performing MRI less restrictive and safer, even with intubated patients. Sadly, MRI availability varies greatly from one hospital to another, and its access is not guaranteed. In our view, this is the primary limitation. Other advantages of the exam should be underlined, mainly that there is no irradiation and no iodine injection, important considerations for IE patients who often have renal failure. Conversely, the innocuousness of gadolinium is currently contested [35, 36]. Furthermore, the impact on the cost of this imaging strategy has not yet been evaluated.

## Conclusion

In infective endocarditis (IE), cerebrovascular complications are a serious consideration in terms of therapeutics and prognosis. In this systemic disorder, brain magnetic resonance imaging (MRI) shows large patterns of lesions: silent ischemic stroke due to microinfarction and microbleeds are the most frequent. Few studies have analyzed the repercussion of brain MRI in the course of IE. Despite the use of a standard definition of the disease, methodologies vary and inhibit comparability. This systematic review has shown limited prospective data. Large studies are also limited. The effect on clinical decision-making and the influence of cerebral microbleeds (CMBs) has not been fully evaluated. Similarly, long-term prognosis assessment is sorely missing. Further studies are necessary to assess the role of microbleeds as potential new imaging clues in IE. At present, there is not a high standard of proof and no evidence for a direct positive impact of brain MRI in IE, and we cannot advise to perform such an exam in all cases of possible or definite IE. However, several arguments support the prompt use of MRI, especially for surgical patients.
